# Renal peritumoral adipose tissue undergoes a browning process and stimulates the expression of epithelial-mesenchymal transition markers in human renal cells

**DOI:** 10.1038/s41598-022-12746-9

**Published:** 2022-05-23

**Authors:** Matías Ferrando, Flavia Alejandra Bruna, Leonardo Rafael Romeo, David Contador, Daiana Lorena Moya-Morales, Flavia Santiano, Leila Zyla, Silvina Gomez, Constanza Matilde Lopez-Fontana, Juan Carlos Calvo, Rubén Walter Carón, Judith Toneatto, Virginia Pistone-Creydt

**Affiliations:** 1 Laboratory of Hormones and Cancer Biology, Centro Científico y Tecnológico Mendoza, Instituto de Medicina y Biología Experimental de Cuyo (IMBECU), Consejo Nacional de Investigaciones Científicas y Técnicas (CONICET), Universidad Nacional de Cuyo, Av. Ruiz Leal w/n, General San Martin Park, CP5500 Mendoza, Argentina; 2 Facultad de Odontología, Centro de Investigaciones Odontológicas (CIO), Universidad Nacional de Cuyo, Mendoza, Argentina; 3 Departamento de Urología y Trasplante Renal, Hospital Español de Mendoza, Mendoza, Argentina; 4 Facultad de Medicina-Clínica Alemana, Centro de Medicina Regenerativa (CMR), Universidad del Desarrollo, Concepción, Chile; 5 Instituto de Biología y Medicina Experimental (IBYME), Consejo Nacional de Investigaciones Científicas y Técnicas (CONICET), Mendoza, Argentina; 6 Departamento de Química Biológica, Facultad de Ciencias Exactas y Naturales, Universidad de Buenos Aires, Buenos Aires, Argentina; 7 Departamento de Fisiología, Facultad de Ciencias Médicas, Universidad Nacional de Cuyo, Mendoza, Argentina

**Keywords:** Cancer, Cell biology, Nephrology, Oncology

## Abstract

Tumor cells can interact with neighboring adipose cells and adipocyte dedifferentiation appears to be an important aspect of tumorigenesis. We evaluated the size of adipocytes in human adipose explants from normal (hRAN) and kidney cancer (hRAT); changes in the expression of WAT and BAT/beige markers in hRAN and hRAT; the expression of epithelial-mesenchymal transition (EMT) cell markers in human kidney tumor (786-O, ACHN and Caki-1); and non-tumor (HK-2) epithelial cell lines incubated with the conditioned media (CMs) of hRAN and hRAT. We observed that hRAT adipocytes showed a significantly minor size compared to hRAN adipocytes. Also, we observed that both Prdm16 and Tbx1 mRNA and the expression of UCP1, TBX1, PPARγ, PCG1α, c/EBPα LAP and c/EBPα LIP was significantly higher in hRAT than hRAN. Finally, we found an increase in vimentin and N-cadherin expression in HK-2 cells incubated for 24 h with hRAT-CMs compared to hRAN- and control-CMs. Furthermore, desmin and N-cadherin expression also increased significantly in 786-O when these cells were incubated with hRAT-CMs compared to the value observed with hRAN- and control-CMs. We observed a significant decrease in E-cadherin expression in the ACHN cell line incubated with hRAT-CMs versus hRAN- and control-CMs. However, we did not observe changes in E-cadherin expression in HK-2, 786-O or Caki-1. The results obtained, together with the results previously published by our group, allow us to conclude that perirenal white adipose tissue browning contributes to tumor development in kidney cancer. In addition, hRAT-CMs increases the expression of mesenchymal markers in renal epithelial cells, which could indicate a regulation of EMT due to this adipose tissue.

## Introduction

In recent years it has been shown that tumor progression also depends on the bidirectional dialogue between tumor epithelial cells and surrounding stromal cells. Among the different types of cells that share a microenvironment with renal epithelial cells, renal adipose tissue is one of the most abundant. Adipose tissue (AT) is mainly responsible for the depository and delivery of energy in response to systemic demands, but also is currently recognized as an endocrine and immunomodulating organ that contributes to human physiology, through both systemic and local-specific functions^1^. Cancer cells and cancer associated adipocytes (CAAs) interact with each other, which promotes the formation of a unique microenvironment stimulating tumor growth and metastasis^2–5^. Therefore, the role of AT on tumor metabolism is becoming increasingly important^6,7^.

Renal cell carcinoma (RCC) is one of the ten most often diagnosed cancers worldwide. Each year over 300,000 new RCC cases are diagnosed and nearly 140,000 patients die of this disease^8^. RCC is a highly metastatic cancer; nearly 30% of all RCC patients have developed metastasis at the time of diagnosis^9^. Clear cell renal cancer (ccRCC) is the most frequent and aggressive type of RCC, and usually represents 80–85% of all RCC. Furthermore, the incidence of RCC has increased globally by 2–3% per year^10^.

Adipocyte dedifferentiation appears to be an important aspect of tumorigenesis^11^. Recent work on breast tissue has shown that the adipocytes close to tumor epithelial cells showed a more rapid depletion of their lipid stores than those that are further away from the tumor^12^; and the adipocytes on the invasive front are smaller than those observed at a distance, suggesting lipolysis^3,13^. Recently we observed that hRAT (human adipose explants from kidney cancer expressed significantly higher amounts of leptin and ObR (leptin receptor) than hRAN (human adipose explants from normal kidney)^5^. In addition, leptin is produced primarily by the adipocytes in white adipose tissue (WAT), where it plays important physiological roles both indirectly (primarily via the nervous system) and directly (in an autocrine action)^14^.

Two main types of adipose tissue have been described: WAT with a classical energy storage function, and brown adipose tissue (BAT) with thermogenic activity^7^. In recent decades, a phenomenon known as ‘browning’ of WAT has been described, which was first reported by Young and colleagues in 1984 ^15^. This process is triggered by the increased gene expression levels of different markers involved in the BAT adipogenic differentiation^16^. Also, leptin has implications in other physiological processes within WAT, such as apoptosis, browning and inflammation^14^. White adipocytes that undergo the browning process are called beige adipocytes. The beige cells of white adipose tissue, as occurs with brown adipocytes, are identified by their multilocular morphology, the high number of mitochondria, and the expression of a set of genes specific to brown fat such as UCP1, PGC1α, and PRDM16. Although brown and beige cells are morphologically similar and both have the ability to perform thermogenesis, they have different origins and responses to certain stimuli^17^. In recent years, a possible browning process of peritumoral mammary adipose tissue in breast cancer has been postulated. Both due to phenotypic modifications of peritumoral adipose tissue, and due to changes in the expression of genes involved in the browning process^7,18–21^. Gantov et al.^21^ demonstrated that beige adipocytes favor tumor progression of both tumor and non-tumor mouse mammary epithelial cell lines. Our group recently demonstrated that hRAT favors tumor progression through soluble factors that it secretes into the microenvironment, unlike hRAN. Thus, hRAT would be able to stimulate a protumorigenic behavior of both tumor and non-tumor human renal epithelial cells^5^. Tumor-induced differentiation to beige/brown adipose tissue is an important contribution to the hypermetabolic state of breast cancer^21^. Therefore, we evaluated changes in the expression of WAT and BAT/beige markers in hRAN and hRAT by different methods.

The extracellular matrix (ECM) is a complex structure made up of different proteins, proteoglycans and polysaccharides. Endocrine activity of adipocytes includes several factors implicated in ECM formation and remodeling^22^. We recently observed that HK-2, 786-O, ACHN and Caki-1 cell lines showed a significant decrease in cell adhesion and increase in cell migration after incubation with hRAT-CMs versus hRAN- or control-CMs^5^. The epithelial-mesenchymal transition (EMT) is a characteristic process of epithelial cells when they acquire migratory capacity. Therefore, in this work we decided to evaluate changes in the expression of EMT cell markers characterized by the expression of epithelial (E-cadherin) and mesenchymal (vimentin, desmin and N-cadherin) markers; in human kidney tumor (786-O, ACHN and Caki-1) and non-tumor (HK-2) epithelial cell lines incubated with the CMs of hRAN and hRAT.

## Results

### The adipocytes that surround the kidney tumor are smaller than the adipocytes that surround a normal kidney

**Figure 1:**
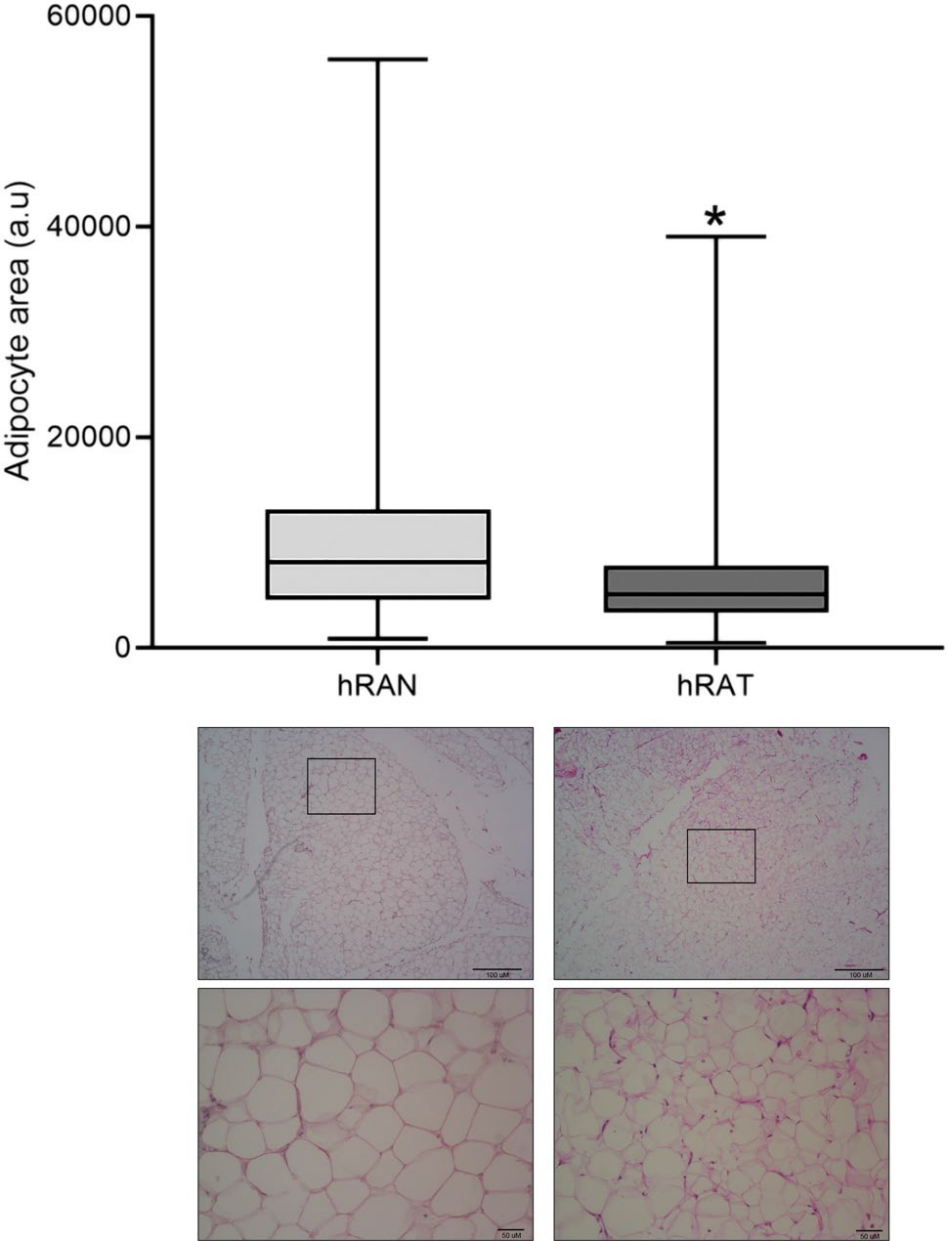
*Microscopic evaluation of different renal adipose tissues.* Slides from adipose tissue samples from hRAN; and tissue samples of tumor kidney attached to the tumor of hRAT. Adipose tissue fragments were cut in paraffin, stained with H&E, and observed under light microscope. Adipocyte size was quantified with Image J software (NIH). The graphic shows the median ± SEM of two independent experiments. *p < 0.05 hRAT attached to the tumor versus hRAN. Magnification: 10X and 40X. Arbitrary units (a.u) represent pixel quantification.

We evaluated the size of adipocytes from different renal adipose tissues. Specifically, we compared the hRAN and hRAT. We observed significant changes in the size of adipocytes in response to the presence of the tumor. The hRAT adipocytes showed a significantly smaller size compared to the hRAN adipocytes (Fig. 1). This change in adipocyte size, together with the increased expression of leptin and its receptor in hRAT versus hRAN^5^, suggest an increase of lipolysis by hRAT adipocytes compared to hRAN adipocytes.

### hRAT showed an increase in gene expression of Prdm16 and TBX1 compared to hRAN

**Figure 2:**
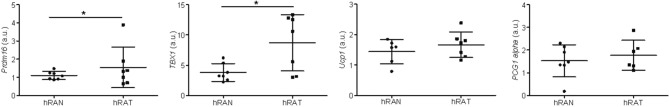
Relative fold expression of Prdm16, TBX1, Ucp1 and PGC1 alpha gene expression from hRAN and hRAT. The mRNA profiles of Prdm16, TBX1, Ucp1 and PGC1 alpha from different adipose tissue were analyzed by qRT-PCR and normalized by their relative ratio to GAPDH. Data are mean ± SEM. GAPDH, glyceraldehyde-3-phosphate dehydrogenase. *p < 0.05.

To evaluate browning of perirenal AT, we measured mRNA levels of *Prdm16*, *TBX1*, *Ucp1* and *PGC1 alpha* in AT from normal and tumor kidney. Increased levels of *Prdm16* and *TBX1* mRNA in hRAT compared to hRAN (Fig. 2, *p* < 0.05) were found. No significant differences were found in Ucp1 and PGC1 alpha in hRAT versus hRAN mRNA.

### UCP1 and PGC1 alpha protein expression increased in hRAT adipocytes compared to hRAN

**Figure 3:**
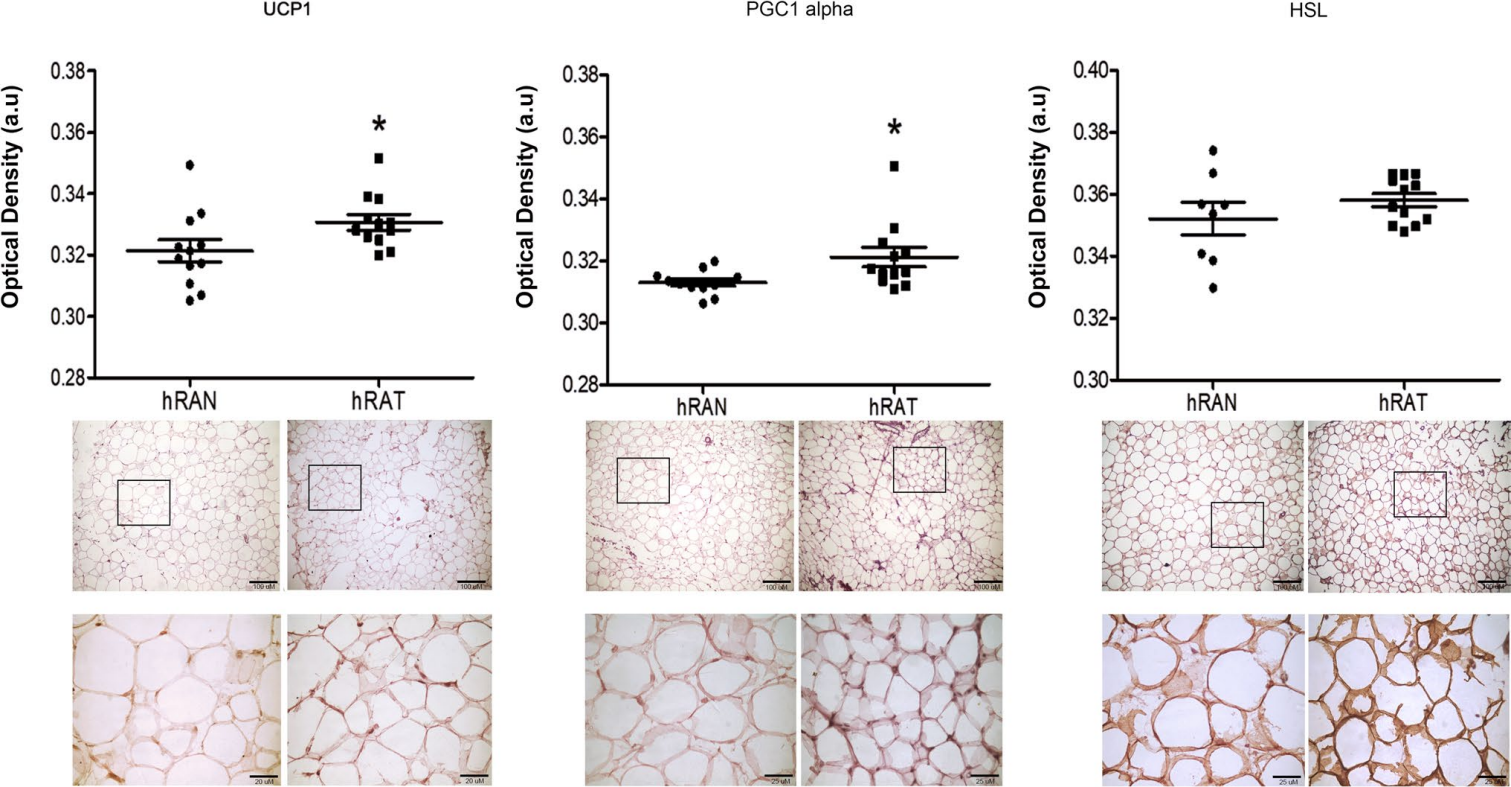
UCP1, PGC1 alpha and HSL expression in histological sections of hRAN and hRAT. UCP1, PGC1 alpha and HSL expression was evaluated by immunohistochemistry in serial cuts of hRAN and hRAT. DAB staining quantification in the three tissue types was performed with Image J software (NIH). Histograms show mean ± SEM of five independent experiments. (a.u.: arbitrary units). *p < 0.01 hRAN versus hRAT. Representative photographs of hRAN- and hRAT-staining. Magnification: 10× and 40×. Arbitrary units (a.u) represent pixel quantification.

We performed immunohistochemistry assays on hRAN and hRAT to measure UCP1, PGC1 alpha and HSL protein levels and localization. UCP1 and PGC1 alpha protein abundance increased in hRAT adipocytes compared to hRAN adipocytes (Fig. 3, *p* < 0.05; negative controls are shown in Supplementary Fig. 1). Also, we observe the multilocular adipocyte morphology in hRAT compared to hRAN (Supplementary Fig. 2).

### The expression of UCP1, TBX1, PPARγ, PCG1 alpha, c/EBPα LAP and c/EBPα LIP was significantly higher in hRAT than hRAN

**Figure 4:**
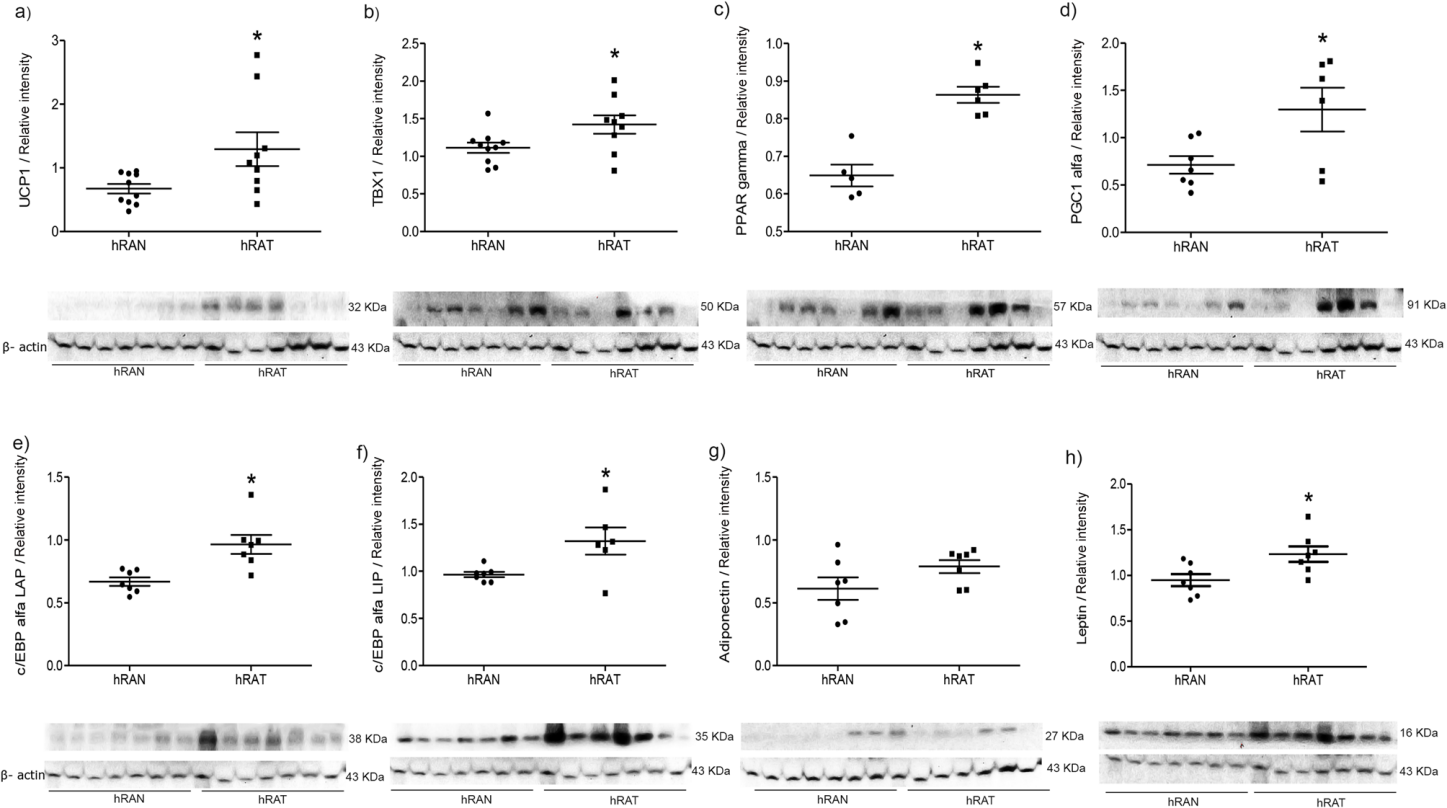
UCP1, TBX1, PPAR gamma, PCG1 alpha, c/EBPα LAP, c/EBPα LIP, adiponectin and leptin in tissue lysate of hRAN and hRAT*.* UCP1, TBX1, PPAR gamma, PCG1 alpha, c/EBPα LAP, c/EBPα LIP, adiponectin and leptin expression were evaluated by Western blot. Images were analyzed by densitometry. Horizontal bars represent the geometric mean of each data set. Vertical bars indicate SEM. *p < 0.05 hRAN versus hRAT.

We evaluated possible changes in the expression of UCP1, TBX1, PPARγ, PCG1α, c/EBPα LAP, c/EBPα LIP, adiponectin and leptin protein in hRAT *versus* hRAN. We observed an increase in UCP1, TBX1, PPARγ, PCG1α, c/EBPα LAP and c/EBPα LIP and leptin expression in hRAT compared to hRAN (*p* < 0.05) (Fig. 4A–F,H). Additionally, we did not observe significant changes in the expression of adiponectin between hRAT and hRAN (Fig. 4G) (Supplementary Fig. 3).

### Soluble factors secreted by hRAT stimulate the expression of mesenchymal markers in tumor and non-tumor human kidney cells

**Figure 5:**
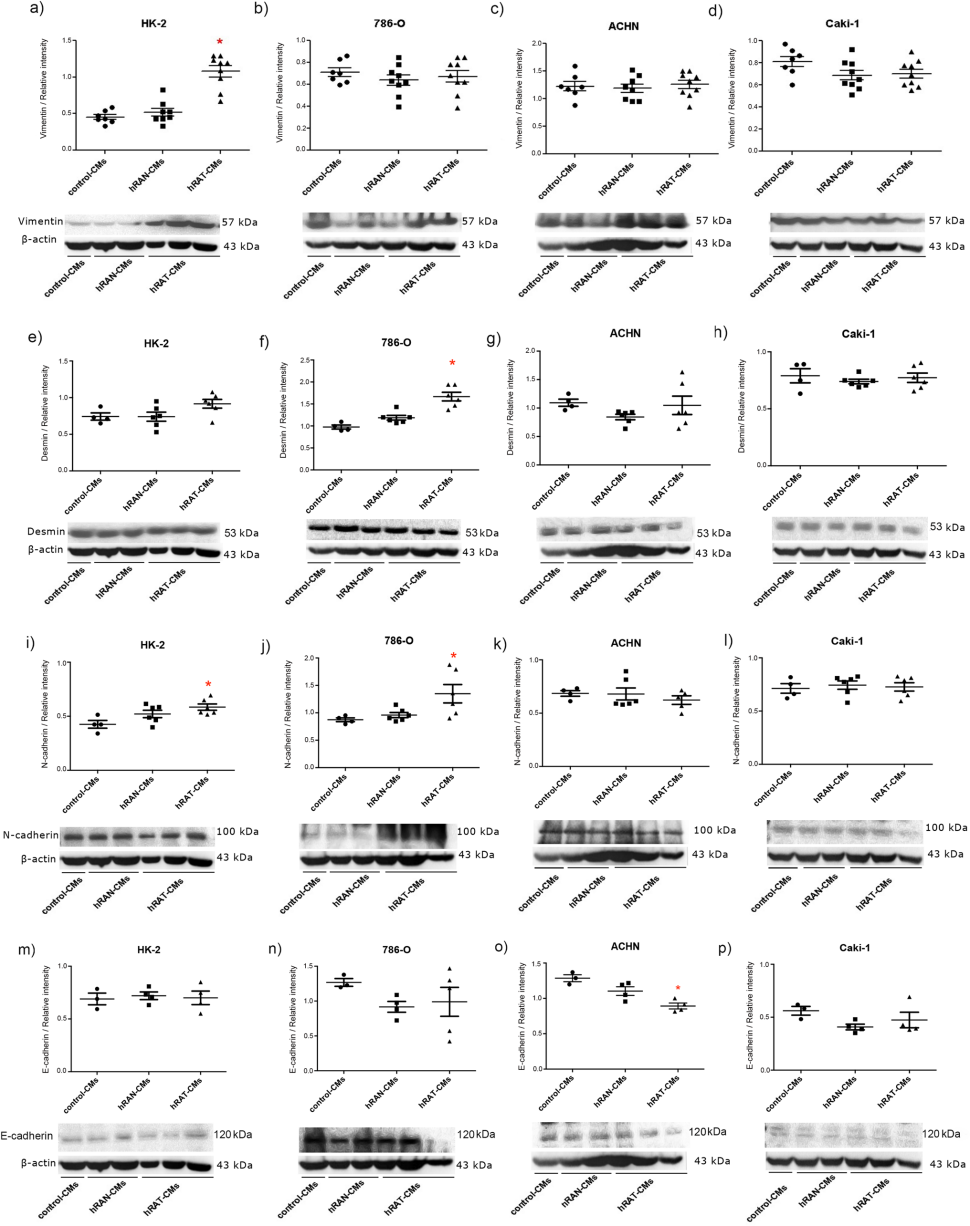
Effect of CMs from hRAN and hRAT on: vimentin (**a**–**d**); desmin (**e**–**h**), N-cadherin (**i**–**l**) and E-cadherin (**m**–**p**) expression was evaluated in HK-2, 786-O, ACHN and Caki-1 cell lines. HK-2, 786-O, ACHN and Caki-1 cells were grown on 6 well plates, incubated for 24 h with the different CMs and then lysed. Expression of the different proteins was measured by Western blot. β-actin was used as internal control. Images were analyzed by densitometry. Horizontal bars represent the geometric mean of each data set. Vertical bars indicate SEM. *p < 0.05 cells incubated with hRAT-CMs versus hRAN- and control-CMs.

We decided to evaluate changes in the expression of EMT cell markers, in human kidney tumor (786-O, ACHN and Caki-1) and non-tumor (HK-2) epithelial cell lines, incubated with the CMs of hRAN and hRAT during 2 h and 24 h. After 2 h post treatment, protein expression changes were not observed among cell lines (data not show on the manuscript, see Supplementary Fig. 4); therefore the experiments were assessed at 24 h. We found an increase in vimentin and N-cadherin expression in HK-2 cells incubated for 24 h with hRAT-CMs compared to hRAN- and control-CMs (*p* < 0.05) (Fig. 5A,I). The same tendency was observed for the expression of desmin, but it was not statistically significant (Fig. 5E). Furthermore, desmin and N-cadherin expression also increased significantly in 786-O when these cells were incubated with hRAT-CMs compared to the value observed with hRAN- and control-CMs (Fig. 5F,J *p* < 0.05). However, no significant changes in vimentin expression were observed in 786-0 cells (Fig. 5B). Likewise, we did not find significant changes in the expression of vimentin, desmin or N-cadherin in the two tumor renal epithelial cell lines originating from metastatic sites (ACHN and Caki-1 cells) (Fig. 5C–D,G–L). Furthermore, we evaluated changes in E-cadherin expression in the four cell lines incubated with hRAT-, hRAN- and control CMs. We observed a significant decrease in this epithelial marker in the ACHN cell line incubated with hRAT-CMs versus hRAN- and control-CMs (Fig. 5O). We did not observe changes in E-cadherin expression in any of the other cell lines tested (Fig. 5 M–P) (Supplementary Fig. 4).

## Discussion

Renal cancer (RCa) is considered to be the fifth most common type of cancer worldwide, having a high mortality rate in both men and women. Tumor development and maintenance of a cancerous phenotype, requires a bidirectional communication between epithelial cells and the stromal environment. Renal adipose tissue is one of the most abundant cell types surrounding renal epithelial cells^23^. Our group has shown that the periprostatic adipose tissue of patients with prostate cancer can regulate tumor behavior, both in early and late stages of the disease^24^. Furthermore, we have worked with adipose tissue fragments from human breast tumors (hATT) and normal breast glands (hATN), from which we obtained the corresponding CMs (hATT-CMs and hATN-CMs). We showed that hATT is capable of stimulating growth and metastatic capacity of mammary tumors by different mechanisms, unlike hATN^3,25,26^. Recently, we demonstrated that renal peritumoral adipose tissue undergoes a process of adaptation to changes locally generated by the tumor^5^. Also, we show that this hRAT is capable of stimulating protumorigenic behavior of renal epithelial cells^5^. We observed that hRAT expressed significantly higher amounts of leptin and ObR, relative to hRAN. It has been shown that leptin has a pro-tumorigenic function and can act by increasing lipolysis^14^. This increase in lipolysis is accompanied by a decrease in the size of adipocytes. Along these lines, our results show a significant decrease in the mean size of hRAT adipocytes with respect to the size of hRAN adipocytes (Fig. 1). The increased expression of both leptin and its receptor, together with the decrease in the size of hRAT adipocytes, allow us to suggest an increase in lipolysis in hRAT adipocytes. The increase in lipolysis would favor an increased availability of energy to the tumor, favoring its development.

Leptin has been found to activate thermogenesis in BAT, increasing UCP1 production. It also exerts browning stimulation in WAT, with a greater number of mitochondria, and expressing UCP1 and other markers of browning, such as PRDM16^27^. Tumor induced differentiation to beige/brown adipose tissue is an important contribution to the hypermetabolic state of breast^21^ and prostate^7^ cancer, but to our knowledge, no previous research has been performed on browning and kidney cancer. However, Jespersen et al*.*^28^ they showed that the perirenal fat in adult humans consist of dormant BAT, and small amounts of adipocytes with a multilocular morphology are present near regions with sympathetic activity. To begin to elucidate this, we evaluated the expression of different markers of WAT and BAT/beige adipocytes in hRAT and hRAN. The browning of WAT is triggered by increased gene expression levels of different markers involved in the BAT adipogenic differentiation, including PPAR gamma or PGC-1 alpha^28^. Furthermore, PPAR gamma induces the expression of C/EBP, which makes this gene a key regulator of WAT differentiation^29,30^. PPAR gamma also induces the expression and production of UCP-1 in these beige adipocytes^31^. UCP1 is a transmembrane protein that uncouples the electron transport chain (ETC) by pumping protons from the intermembrane space back into the mitochondrial matrix, thereby generating heat rather than ATP^32^. As well, PRDM16 is a master regulator gene of brown adipocyte differentiation and TBX1 beige adipocyte marker expression^18^. We observed that both Prdm16 and Tbx1 genes and protein expression of UCP1, TBX1, PPAR gamma, PCG1 alpha, c/EBPα LAP and c/EBPα LIP was significantly higher in hRAT than hRAN (Figs. 2, 3, 4). Considering our results together with those published by Jespersen et al*.*^28^, the increased browning found in hRAT compared to hRAN could be due to both stimulation of brown/beige adipogenesis of progenitors and/or transdifferentiation of white to brown/beige adipocytes. However, the human adipose explants from kidney samples used to perform the experiments were taken from regions far from sources of local sympathetic activity, that is, from remote areas in which reservoirs of a latent BAT state have been identified. This is the first work that demonstrates a transdifferentiation of WAT adipocytes in the human perirenal AT that surrounds a renal tumor, since we observed an increase in the amount of beige adipose tissue in hRAT, as opposed to hRAN. In light of these results, together with those previously described^5^, we postulate that the renal tumor would be regulating the differentiation of the surrounding WAT and its browning. The browning process might have a role in the development of kidney tumors. Currently, and to deepen our understanding of this possible regulation, we set out to study changes in WAT and BAT/beige adipocytes markers in hRAN fragments incubated with MCs from tumor kidney cell lines. Preliminary results (not yet published) allow us to observe an increase in the expression of BAT/beige adipocyte markers in hRAN, which would support the hypothesis that the renal tumor would be stimulating browning of the surrounding AT.

Finally, we evaluated the ability of hRAT- and hRAN-CMs to produce changes in the expression of EMT cell markers; in human kidney tumor and non-tumor epithelial cell lines incubated with MCs of hRAN and hRAT during 2 h and 24 h. After 2 h post treatment, no protein expression changes were observed among the cell lines (data not show on the manuscript, see Supplementary Fig. 3); therefore the experiments were assessed at 24 h. In accordance with our previous results, where we showed that the cell lines decrease in cell adhesion and increase in cell migration after incubation with hRAT-CMs versus hRAN- or control-CMs, in the current work we found an increase in vimentin and N-cadherin expression in HK-2 cell, and desmin and N-cadherin expression in 786-O cells incubated with hRAT-CMs compared to hRAN- and control-CMs. Several published works have demonstrated the ability of HK-2 cells to undergo EMT against different stimuli^33–35^. Furthermore, we observed a significant decrease in this epithelial marker in ACHN cell line incubated with hRAT-CMs versus hRAN- and control-CMs. However, we did not observe changes in the expression of E-cadherin (epithelial marker) in any of the cell lines used. Increased expression of cellular mesenchymal markers was observed both in the non-tumor cell line (HK-2) and in the cell line from the primary tumor (786-O), while no changes were observed in the two cell lines from metastatic sites (ACHN and Caki-1). This could be indicating that factors secreted by renal peritumoral AT facilitate the acquisition of a mesenchymal phenotype in cells that have not yet migrated (HK-2 and 786-O). On the other hand, we did not observe changes in the expression of E-cadherin in our experimental condition. This result could be due to partial and not total EMT, which has already been described in cancer^36^. Likewise, it has been seen that leptin is capable of stimulating EMT. Previously we showed that hRAT expressed significantly higher amounts of leptin and ObR, relative to hRAN^5^. It is known that leptin induces a fibroblastoid morphology evidenced by the decrease in the expression of epithelial markers (occludin, E-cadherin) and an increase in mesenchymal markers (fibronectin, N-cadherin, and vimentin) in breast cancer^37^. Therefore, we postulate that this adipokine could be involved in increasing the expression of EMT markers. Future experiments should use leptin neutralization antibodies in the conditioned media, to confirm our hypothesis about the importance of this adipokine in the observed effects.

This is the first report demonstrating that hRAT presents beige/brown adipocyte characteristics, unlike hRAN. This browning process could be stimulated by the kidney tumor itself, and play an important role in renal tumor development. Furthermore, we demonstrate that hRAT produces soluble factors that facilitate the acquisition of a mesenchymal phenotype in cells that have not yet migrated.

## Methods

### Reagents

Reagents were from Sigma Chemical Co (St. Louis, MO, USA), tissue culture flasks, dishes, and multi-well plates were from Falcon Orange Scientific (Graignette Business Park, Belgium), and culture media from both tissue and cell lines and supplements were from Gibco BRL (Carlsbad, CA, USA)^3–5^.

### Sample collection and handling

Patients with suspected kidney cancer or healthy kidney donors were enrolled. After signing the informed consent, subjects were interviewed using a standard questionnaire that requested information about socio-demographic, medical, and lifestyle factors. Human adipose tissue explants from cancerous (hRAT; n = 23) kidneys were obtained from patients to whom a partial or total (tumor) nephrectomy was performed. Human adipose tissue explants from normal kidneys (hRAN; n = 19), were obtained from live kidney donors who had not received previous chemotherapy or radiotherapy treatment. Perirenal adipose tissue biopsies were taken as follows: (1) in the case of living kidney donors, the adipose tissue fragment was taken 1 cm away from the kidney. From the middle zone (middle pole); (2) in the case of patients with renal tumor, the fragment of adipose tissue was taken 1 cm from the kidney, also taking into account the location of the tumor. Trying, in all cases, to take the sample 1 cm from the location of the tumor, getting as close as possible to the middle zone. In all cases, biopsies were taken distant from the adrenal gland (sources of norepinephrine).

The median body mass index (BMI) of patients was: 26.8 kg/m^2^ for patients with renal tumor (hRAT), and 24.9 kg/m^2^ for living kidney donors (hRAN). BMI (kg/m2) was calculated as weight (kg) divided by height (m) squared.

Samples were transported in PBS and processed immediately. On average, 2 h elapsed from the acquisition of the surgical sample until it was processed under a sterile laminar flow hood. The project was approved by the Medical School’s ethics committee (Universidad Nacional de Cuyo, Argentina) according to the Declaration of Helsinki of experimentation with human subjects. All patients gave their informed consent to undergo tissue harvesting for this research^5^.

### Gene expression by RT-qPCR analysis

Total RNA was extracted from 100 mg of tissue using Trizol reagent (Invitrogen, Carlsbad, CA, USA) and quantified according to its absorbance at 260 nm (NanoDrop 2000, Thermo Scientific, Wilmington, USA). Contaminating genomic DNA was degraded with DNAse RQ1 (Promega, Madison, USA), cDNA was synthesized from one microgram of total RNA using 300 pmol oligo-dT primers, 10 mM dNTP (Thermo Scientific, Wilmington, USA) and 200U M-MLV reverse transcriptase (Promega, Madison, USA). Real-time qPCR was performed in a final volume of 20 uL containing 50 ng cDNA, 3 mM MgCl2, PCR LightCycler-DNA Master SYBRGreen reaction mix (Roche, Indianapolis, USA) and 0.5 mM of each specific primers (Table 1). Amplification was performed using a LightCycler thermocycler (Roche, Indianapolis, USA). Controls without reverse transcription were included to ensure that amplifications were from mRNA and not from genomic DNA. Amplicons were characterized according to their melting temperature and size. The mRNA level of each target gene was calculated using the 2ΔΔCt method and normalized against the mRNA of GAPDH^4,5^.

**Table 1:** primer pair sequence are shown for the Forward (F) and Reverse (R) primers used to measure mRNA abundance by RT-qPCR.

Gen	Forward (5′–3′)	Reverse (5′–3′)	Ct	Size (pb)	TM (°C)	Gene bank
*Prdm16*	GGCAAACGCTTCGAATGTGA	ACCGTGCTGTGGATATGCTT	35	173	94	NM_199454.2
*Tbx1*	ACGACAACGGCCACATTATTC	CCTCGGCATATTTTCTCTATCT	35	102	85	AF012131.1
*Ucp1*	GCAGGGAAAGAAACAGCACCTA	TCCCTTTCCAAAGACCCGTCAA	35	217	86	NM_021833.4
*Pgc1 alpha*	ACCAGCCAACACTCAGCTAA	AGGGACGTCTTTGTGGCTTT	35	170	83	NM_013261.3
*GAPDH*	GGAGCGAGATCCCTCCAAAAT	GGCTGTTGTCATACTTCTCATGG	40	197	89	NM_002046.3

### H&E staining

Tissues (hRAT and hRAN) were fixed in 4% formaldehyde and embedded in paraffin. They were afterwards cut into sections of 5 μm thickness with a microtome, deparaffinized and stained with hematoxylin–eosin (H&E). Images were taken with a Nikon Eclipse E200 Microscope fitted with a digital still camera Micrometric SE Premium (Nikon Corp., Japan) at 100× magnification. Adipocyte area quantification (measuring adipocyte perimeter) in the three tissue types was performed in 8–10 fields of each preparation as mentioned above^5^.

### Immunohistochemistry

10 µm serial cuts were performed on the same tissue samples embedded in paraffin used for H&E staining. UCP1, PGC1 alpha and HSL expression were studied by means of immunohistochemistry. Briefly, hRAN and hRAT microtome slides were first deparaffinized, and then a heat-mediated antigen retrieval, endogenous peroxidase blocking and nonspecific tissue blocking were performed. Slides were then incubated with the different primary antibodies (Anti-UCP-1. SIGMA U6382. Dilution of 1:500; Anti-PGC1 alpha. Abcam ab54481. Dilution of 1:300; and Anti-HSL. Abcam ab45422. Dilution of 1:300) at 4 °C. And after that with an anti-rabbit biotinylated secondary IgG antibody. Finally, slides were incubated with peroxidase-conjugated streptavidin. Peroxidase reaction was performed with chromogen 3,3′-diaminobenzidine (DAB) (DAKO LSAB + Kit, HRP). Hematoxylin counterstaining was performed. Serial cuts incubated in the absence of the primary antibody were used as negative controls. Images were taken with a Nikon Eclipse E200 Microscope fitted with a Micrometric SE Premium (Nikon Corp., Japan) digital still camera at 10× and 40× magnification. DAB staining quantification in the three tissue types was performed in 5 fields of each preparation as mentioned above^3–5^.

### Preparation of conditioned media (CMs) from hRAN and hRAT

Adipose tissues were washed with cold PBS 1X (Gibco, USA and weighed. hRAN or hRAT were plated in culture flasks with M199 culture medium (Invitrogen™; 1 g tissue/10 ml M199), and incubated for 1 h at 37 °C in 5% CO_2_. After that, the medium was removed and replaced with fresh medium and the tissues were incubated for 24 h. Subsequently, the supernatant was collected and filtered using filters with 0.22 µm membranes. Then, supernatants were aliquoted into 1 ml fractions and immediately stored at − 80 °C. The control-CMs were obtained from the collection of the serum-free M199 medium after 24 h of incubation in a culture flask at 37 °C in 5% CO_2_^5^.

### Treatment with hRAN- and hRAT-CMs

In order to study EMT protein expression of tumor (786-O, ACHN and Caki-1) and non-tumor (HK-2) human renal epithelial cell lines, MCs collected were diluted 1:1 in DMEM-F12 (Invitrogen, UK) 2% fetal bovine serum (FBS; 1% FBS final concentration) and the cells were incubated for 2 h (data not shown) and 24 h with the diluted CMs. The experiments were performed with equal volumes of hRAN- and hRAT-CMs. The concentration of total protein in those volumes was quantified using Pearce BCA protein assay kit (Thermo Scientific)^4,5^.

### Culture of tumor and non-tumor renal epithelial cell lines

Tumor (786-O, ACHN and Caki-1) and non-tumor (HK-2) human renal epithelial immortalized cell lines were used. 786-O (ATCC® CRL1932™), ACHN (ATCC® CRL1611™), Caki-1 (ATCC® HTB46™) and HK-2 (ATCC® CRL2190™) were obtained from the American Type Culture Collection (ATCC, Rockville, MD). 786-O is a line derived from a primary clear cell adenocarcinoma (primary tumor); and both ACHN and Caki-1 are lines derived from metastatic sites (pleural effusion and skin respectively). The four cell lines were cultured in DMEM-F12 medium with 10% FBS and 2 µg/ml insulin; and were maintained at 37 °C in 5% CO_2_^5^.

### Preparation of cell lysates from renal epithelial cells after incubation with hRAN-, hRAT- or control-CMs

786-O, ACHN, Caki-1 and HK-2 cells were seeded in six-well plates in DMEM-F12 complete medium. When cells reached 75–80% confluence, the medium was aspirated and cells were washed twice with PBS 1X (Gibco, USA). Then, cells were incubated at 37 °C for 24 h either with hRAN-, hRAT- or control-CMs (50% CM, 50% DMEM-F12 2% FSB). Cells were lysed with Ripa buffer (Tris 10 mM pH 7,5; NaCl 150 mM; sodium vanadate 2 mM; sodium deoxycholate; SDS 0,1%; igepal 1%; protease inhibitors), pelleted by centrifugation at 4 °C and stored at − 80 °C^4,5^.

### Western blot analysis

In order to evaluate protein expression levels, Western blots were performed. UCP1, TBX1, PPARγ, PCG1 alpha, c/EBPα LAP, c/EBPα LIP, adiponectin, leptin, vimentin, desmin, N-cadherin and E-cadherin were measured after incubation of the epithelial cell lines with the different CMs obtained. The cells were lysed with Ripa buffer. Total protein in samples was quantified using the Pierce BCA protein assay kit (Thermo Scientific). Proteins were separated in a SDS-PAGE 12 gel, and electrotransferred to a PVDF membrane (Bio-Rad, USA). The membrane was later blocked with human serum albumin (Sigma-Aldrich, 0055 K) and then incubated with the different antibodies ON at 4 °C. The membranes were later washed, and incubated with proper secondary antibodies conjugated with biotione, and subsequently the signal was amplified with streptavidin. Antibody complexes were visualized by means of chemiluminescence (ECL; GE Helathcare). Membrane exposed images were obtained with the Chemidoc MP system (Bio-Rad, USA) and bands were quantified by densitometry using the FIJI Image processing package (NIH, USA)^22^. In cell extracts, β-actin level in samples was used to determine that equal quantities of proteins were loaded in the gel ^3–5^.

### Statistical methods

The statistical significance between different experimental conditions was evaluated by *t-*test or one-way ANOVA. Tukey´s post-hoc tests were performed within each individual treatment. The results are presented as mean ± SEM. Results were considered significant at *p* < 0.05.

## Supplementary Information

Supplementary Information 1.

Supplementary Information 2.

Supplementary Information 3.

Supplementary Information 4.

Supplementary Information 5.
