# Supplementary figures and images for: Renal peritumoral adipose tissue undergoes a browning process and stimulates the expression of epithelial-mesenchymal transition markers in human renal cells
Source: Sci Rep. 2022 May 23;12:8687. doi: 10.1038/s41598-022-12746-9 (PMC9127098; doi:10.1038/s41598-022-12746-9)

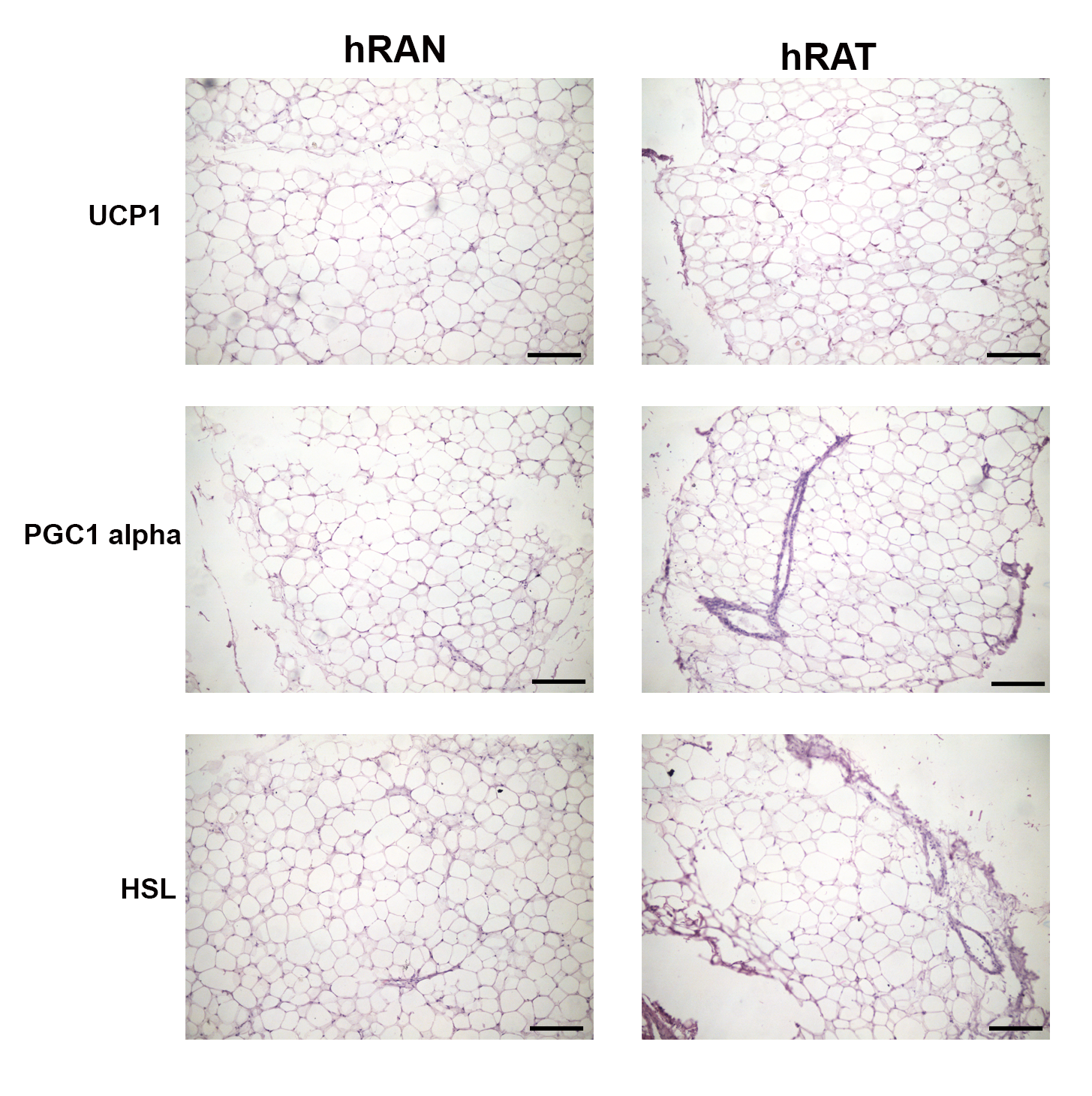

Supplement: Supplementary file 1 — Supplementary Information 1. [file 41598_2022_12746_MOESM1_ESM.tif]

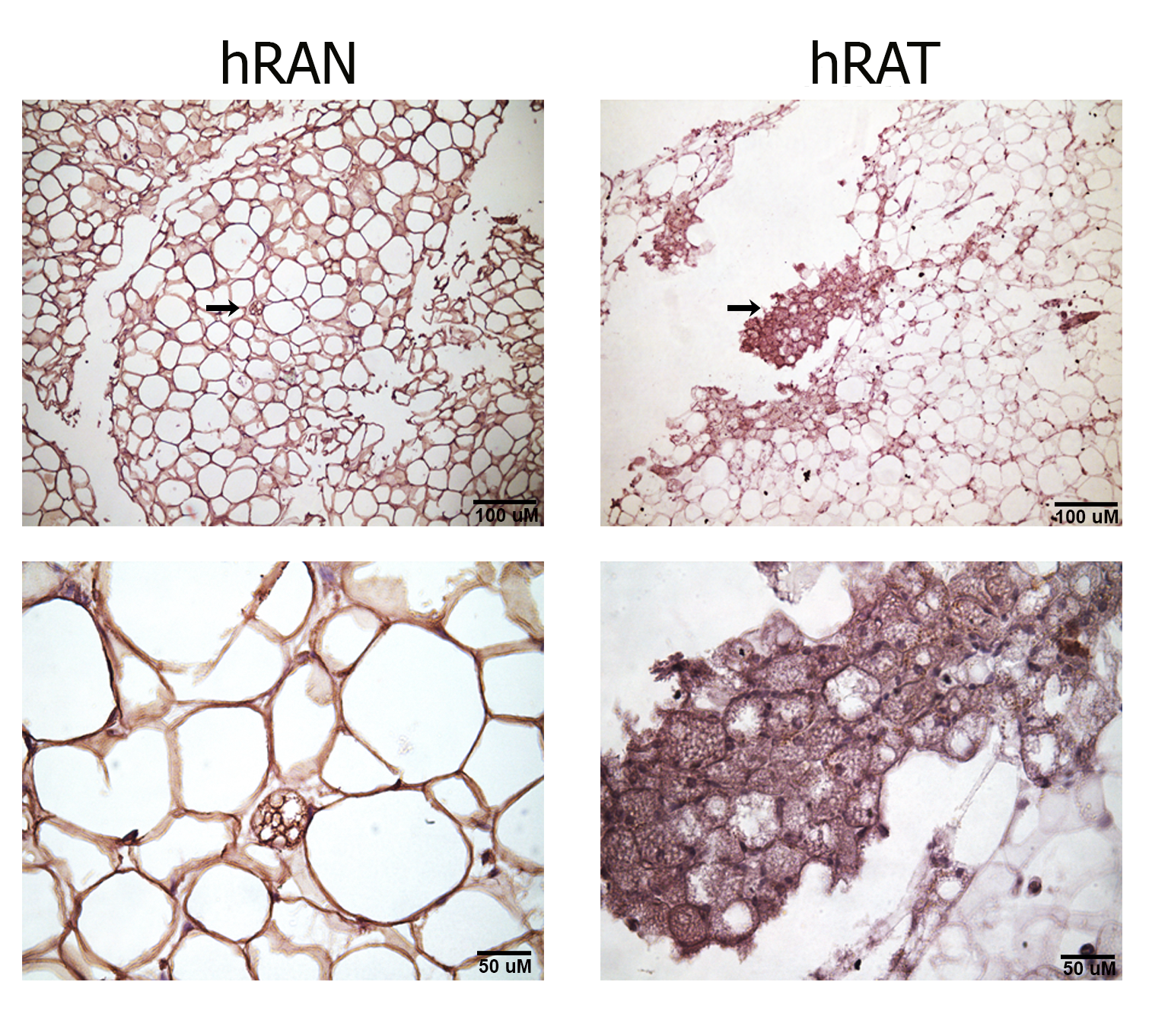

Supplement: Supplementary file 2 — Supplementary Information 2. [file 41598_2022_12746_MOESM2_ESM.tif]

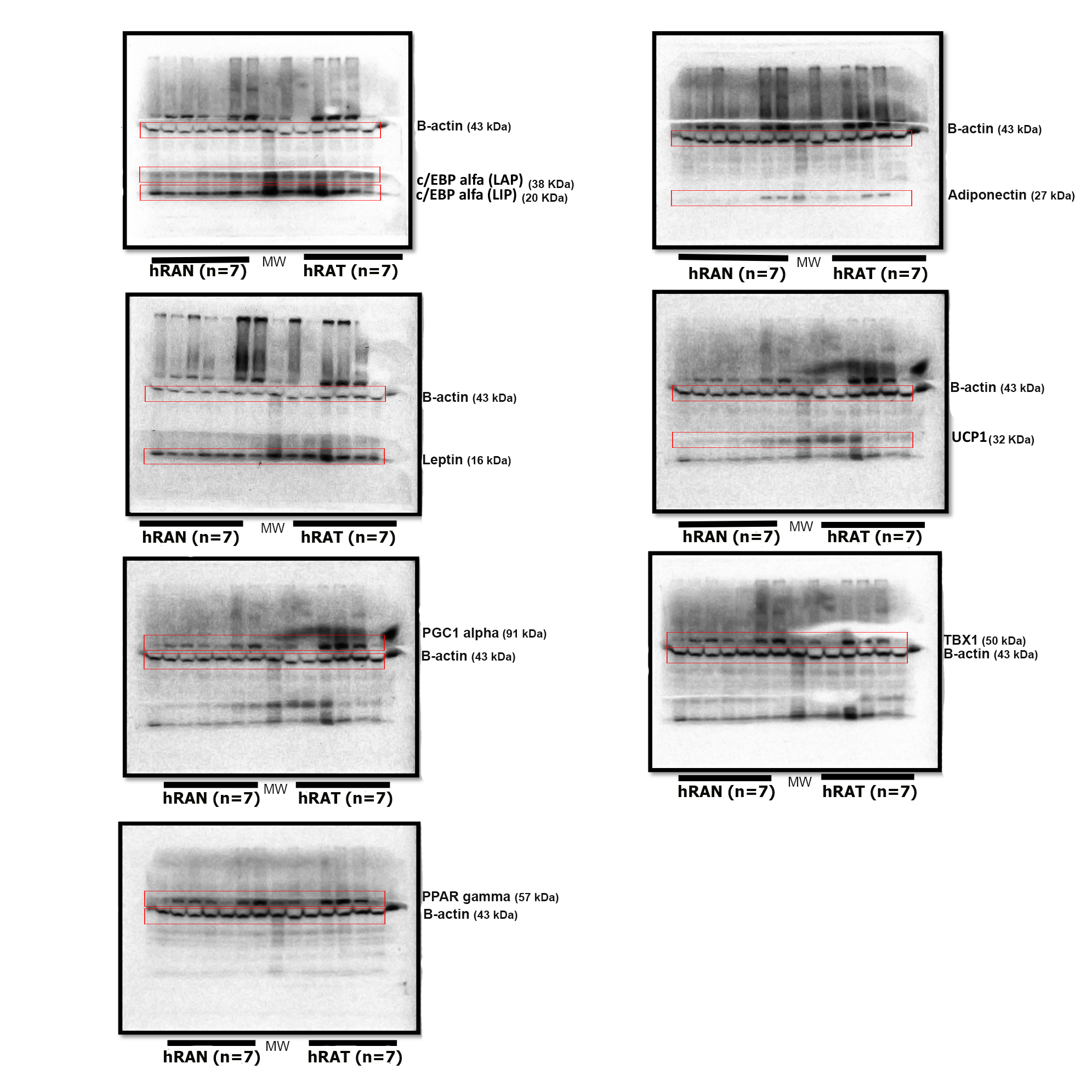

Supplement: Supplementary file 3 — Supplementary Information 3. [file 41598_2022_12746_MOESM3_ESM.tif]

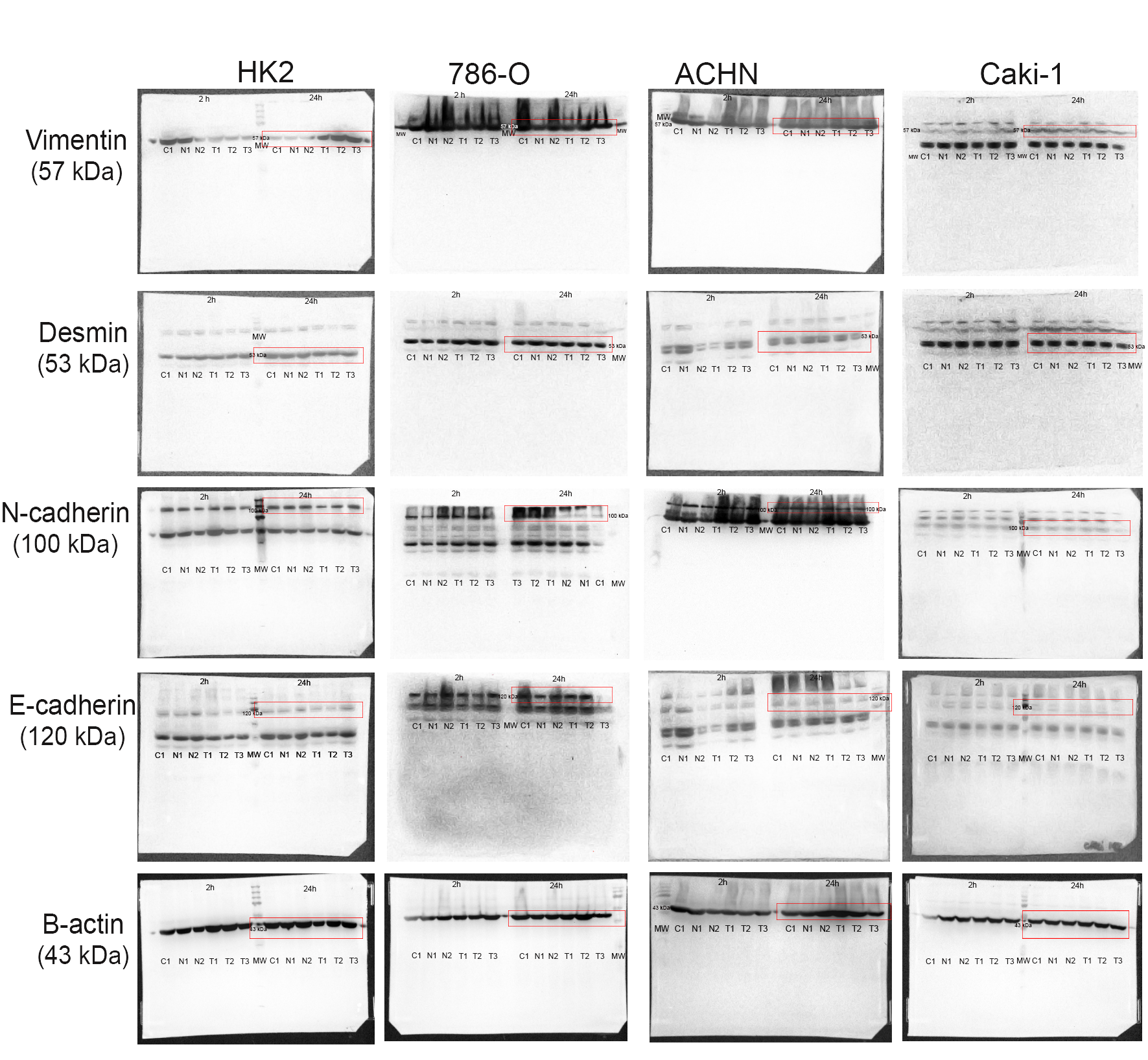

Supplement: Supplementary file 4 — Supplementary Information 4. [file 41598_2022_12746_MOESM4_ESM.tif]
