# Supplementary material for: Renal peritumoral adipose tissue undergoes a browning process and stimulates the expression of epithelial-mesenchymal transition markers in human renal cells
Source: Sci Rep. 2022 May 23;12:8687. doi: 10.1038/s41598-022-12746-9 (PMC9127098; doi:10.1038/s41598-022-12746-9)
Supplement: Supplementary file 5 — Supplementary Information 5. [file 41598_2022_12746_MOESM5_ESM.docx]

**Supplementary Figure 1:** Negative controls (secondary antibody only ones) of each of the antibodies used in IHC. Complementary figure of Figure 3 of the paper. Representative images of hRAN- and hRAT-staining. Magnification: 10X.

**Supplementary Figure 2:** Representative images of hRAT and hRAN. Can be observed the multilocular adipocyte morphology in hRAT compared to hRAN.

**Supplementary Figure 3:** Representative and original complete membranes of Western blots made from the lysis of hRAT and hRAN fragments. Complementary figure of Figure 4 of the paper. The red box indicates the area of the gel that was quantified and is shown in the Figure 4.

**Supplementary Figure 4:** Representative and original membrane fragments of Western blots made from the lysis of HK-2, 786-O, ACHN and Caki-1 cell lines, incubated with hRAT-CMs vs. hRAN- and control-CMs. The lane corresponding to the molecular weight marker is conserved in each membrane fragment. Complementary figure of Figure 5 of the paper. The red box indicates the area of the gel that was quantified and is shown in the Figure 5.
